# Surgical Reintervention for Complex, Multivalvular Rheumatic Heart Disease

**DOI:** 10.1016/j.jaccas.2025.104356

**Published:** 2025-10-22

**Authors:** Kathleen R. Khan, Christopher R. Burke, Jill M. Steiner

**Affiliations:** a Division of Cardiology, Department of Surgery, University of Washington School of Medicine, Seattle, Washington, USA; b Division of Cardiothoracic Surgery, University of Washington School of Medicine, Seattle, Washington, USA

**Keywords:** aortic valve, mitral valve, rheumatic heart disease, tricuspid valve, valve replacement

## Abstract

**Background:**

Multivalve intervention carries elevated risk, particularly after prior valve surgeries.

**Case Summary:**

A 52-year-old woman with rheumatic heart disease presented with progressive dyspnea. Multimodality imaging revealed severe bioprosthetic aortic valve regurgitation, severe bioprosthetic mitral valve stenosis, and severe tricuspid valve regurgitation. Although she was at increased surgical risk, the small size of her aortic valve prosthesis limited transcatheter options. She elected to undergo cardiac surgery, which included bioprosthetic Bentall and Commando procedures as well as tricuspid valve repair. Postoperative course was complicated by right ventricular dysfunction with prolonged hospitalization; however, she recovered and was discharged home.

**Discussion:**

This case demonstrates the challenges of multivalve intervention, despite comprehensive preparation including shared decision making and heart valve team planning.

**Take-Home Messages:**

Multidisciplinary planning for reintervention in multivalve disease can be complex without a single best solution. Shared decision making can help patients understand risks and make decisions aligned with their values.

## Graphical Abstract

**Figure:**
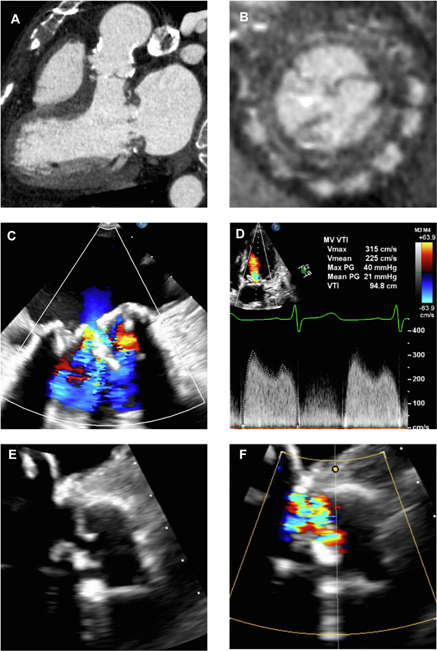

## History of Presentation

A 52-year-old woman presented to the cardiology clinic for follow-up, noting fatigue and worse exertional tolerance with housework. She had a history of rheumatic heart disease affecting the aortic and mitral valves. At age 21, she underwent surgical bioprosthetic aortic valve replacement (AVR) for severe stenosis. At age 38, in the setting of severe bioprosthetic aortic valve and mitral valve regurgitation, she underwent repeat AVR with a 21-mm Freestyle (Medtronic) aortic root bioprosthesis and mitral valve replacement with a 29-mm Mosaic (Medtronic) bioprosthesis. Bioprosthetic valves were chosen per patient preference during her childbearing years.

**Take-Home Messages**

• Multidisciplinary planning for reintervention in multivalve disease can be complicated without a single correct answer.
• Shared decision making can help patients understand the risks and benefits of potential treatments and make decisions aligned with their values.

On examination, vital signs were within normal limits, and she was well appearing. Cardiac examination was notable for a systolic murmur best heard at the lower left sternal border. Lungs were clear, abdomen was benign, and she had no peripheral edema.

Transthoracic echocardiography (TTE) revealed normal biventricular function and elevated gradients across both bioprosthetic valves. Mitral stenosis was worse with mean gradient of 13 mm Hg at heart rate 80 beats/min from 8 mm Hg prior. Aortic stenosis was mild with peak antegrade velocity 2.6 m/s compared with 1.6 m/s prior. No aortic regurgitation (AR) was visible. Estimated pulmonary artery systolic pressure was elevated at 39 to 44 mm Hg. The Freestyle prosthesis was heavily calcified.

## Past Medical History

Additional history included atrial flutter ablation, paroxysmal atrial fibrillation diagnosed after her most recent surgery (on apixaban), dual-chamber pacemaker for sinus node dysfunction, hyperlipidemia, hypertension, and diabetes mellitus complicated by stage III chronic kidney disease.

## Differential Diagnosis

Differential diagnoses for her symptoms included prosthetic valve degeneration and valve thrombosis. Endocarditis was considered; however, she denied any infectious symptoms. Ischemic disease was thought less likely given a recent normal stress test.

## Investigations

**Figure 1:**
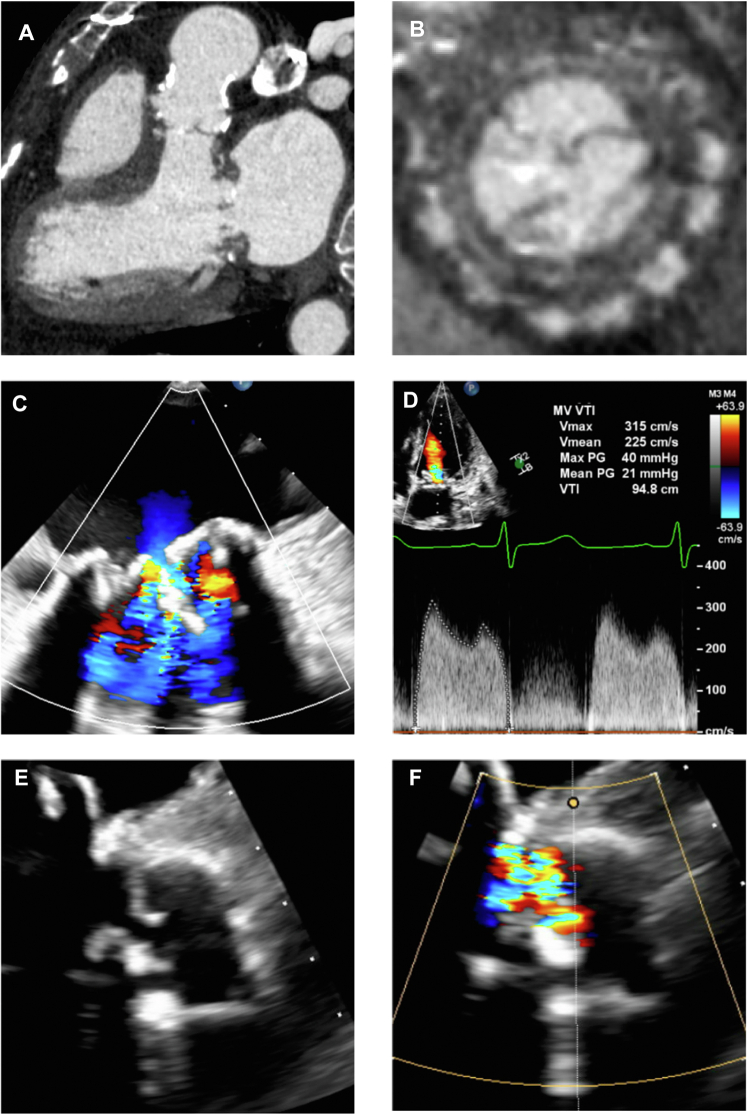
Multimodality Imaging Evaluation Cardiac computed tomography showed extensive calcification of the Freestyle aortic root and aortic valve bioprosthesis (A) and bioprosthetic mitral valve (B), without evidence of valve thrombosis. Transesophageal echocardiography showed bioprosthetic mitral valve leaflet thickening with significantly decreased excursion (C) and mild to moderate regurgitation. Before surgery, transthoracic echocardiography showed severe bioprosthetic mitral valve stenosis with diastolic mean gradient of 21 mm Hg (D). Transesophageal echocardiography also revealed severe bioprosthetic aortic valve regurgitation secondary to a flail anterior cusp (E, F).

Laboratory evaluation was notable for mildly elevated B-type natriuretic peptide of 174 pg/mL (reference range <101 pg/mL). Creatinine was 1.62 mg/dL (reference range 0.38-1.02 mg/dL), similar to prior values. Blood cultures were negative. Pacemaker interrogation showed no tachyarrhythmias. Apixaban was switched empirically to warfarin, and a cardiac computed tomography scan was obtained to rule out valve thrombosis, which showed mitral calcification most consistent with calcific degeneration (Figures 1A and 1B, Video 1).

Transesophageal echocardiography (TEE) showed thickened and restricted bioprosthetic mitral valve leaflets with a diastolic mean gradient of 6 mm Hg at heart rate 50 beats/min and moderate regurgitation (Figure 1C, Video 2). Severe AR was noted with a flail anterior cusp, not seen on prior studies (Figures 1E and 1F, Videos 3 and 4). Tricuspid regurgitation (TR) was severe. She had normal left and right ventricular size and systolic function.

Coronary angiography revealed mild nonobstructive disease. Right heart catheterization was notable for elevated right- and left-sided filling pressures, elevated mean pulmonary artery pressure of 40 mm Hg, pulmonary vascular resistance of 3.25 Woods units, and transpulmonary gradient of 9 mm Hg, indicating postcapillary pulmonary hypertension. Cardiac index was reduced at 1.8 L/min/m^2^.

Repeat TTE for worsening symptoms showed moderate left ventricular dilation with an end-diastolic volume index of 86 mL/m^2^ and normal systolic function with ejection fraction 56%. Right ventricular (RV) systolic function was mildly reduced with tricuspid annular plane systolic excursion of 10 mm and RV tissue Doppler velocity of 9.4 cm/s. Mitral valve stenosis was worse with mean gradient 21 mm Hg at 76 beats/min (Figure 1D). Bioprosthetic AR and TR remained severe. Pulmonary artery systolic pressure was severely elevated, estimated at 87 to 92 mm Hg.

## Management

The patient continued to experience progressive heart failure symptoms and renal dysfunction despite up-titration in diuretics. She was able to walk only a few steps and was unable to lie flat.

The case was discussed at a multidisciplinary conference. There was concern that she would be high risk for surgical intervention given her prior surgeries, comorbidities, and invasive hemodynamics. Transcatheter intervention was also proposed; however, this was thought to be suboptimal as it would require multiple staged procedures, and valve-in-valve transcatheter AVR would leave limited options for future reintervention given the small size of her bioprosthesis. Options were discussed with the patient, including the risk of worsening renal function and dialysis. Following the discussion, she elected surgical intervention.

The patient was admitted for cardiac surgery, which included bioprosthetic AVR and root replacement (Bentall procedure) with a 23-mm Konect Resilia valved conduit (Edwards Lifesciences), mitral valve replacement with a 29-mm Epic bioprosthetic valve with reconstruction of the aortomitral fibrous body (Commando procedure), tricuspid valve repair, removal of her intravenous pacemaker leads, and placement of epicardial pacing wires. The operative course was complicated by severe calcification of the Freestyle graft and small coronary buttons, aortic root bleeding, and severe RV dysfunction refractory to inotropic support. TEE following cardiopulmonary bypass wean showed that RV fractional area change was severely reduced at 16%. She was placed on venoarterial extracorporeal membrane oxygenation support and transported to the cardiac catheterization laboratory where ostial right coronary artery stenosis was treated with a 3.5 × 28 mm drug-eluting stent.

She had a prolonged intensive care course, with central venoarterial extracorporeal membrane oxygenation transitioned to right ventricular assist device. Although she was able to be weaned from mechanical circulatory support, she remained on inotropic support until postoperative day 24. Additional complications included ventilator-associated pneumonia, acute renal injury requiring dialysis, epicardial pacemaker lead dysfunction requiring leadless device placement, and surgical debridement of a sacral wound.

## Outcome and Follow-Up

After a 1.5-month hospitalization, the patient was discharged home with physical/occupational therapy. At the time of discharge, renal function had improved to baseline. She also had follow-up scheduled with electrophysiology, nephrology, endocrinology, and wound care subspecialists.

At her 1-month posthospitalization appointment, she was recovering well. She had normal biventricular and prosthetic valve function. At 6 months, she was walking for 30 minutes a day, although still needing daily diuretics.

## Discussion

This was a challenging case of a patient with rheumatic heart disease and 2 prior cardiac surgeries who developed rapid degeneration of bioprosthetic aortic and mitral valves as well as severe TR. Her presentation highlights how it can be difficult to differentiate the primary driver of symptoms in patients with multiple valvular abnormalities.

As stated in the most recent American College of Cardiology/American Heart Association Guidelines, a comprehensive approach to imaging and hemodynamic evaluation is required in mixed valve disease, as the typical methods for estimating severity may be limited.^1^ For example, the severity of this patient's aortic valve pathology could have been underestimated owing to reduced left ventricular filling from mitral stenosis. Additionally, detection of bioprosthetic mitral regurgitation is particularly difficult on TTE due to acoustic shadowing, so TEE is often needed for diagnosis.^2^

A multidisciplinary heart valve team meeting is also emphasized in the guidelines as it is key for discussing advantages and risks of each approach.^1^^,^^3^ In this case, transcatheter options would have required multiple staged procedures, and there was concern about feasibility with her small aortic bioprosthesis as well as low coronary implants, risking coronary obstruction and paucity of options for future interventions. Transcatheter tricuspid valve intervention would also be challenging with the presence of a right ventricular pacemaker lead. Although she had increased risk with a triple-valve surgery and third sternotomy, surgery was thought to offer the most comprehensive solution for her complex anatomy. Together, this discussion informed shared decision making with the patient, and she decided to proceed with surgery.

This case also highlights important considerations in choice of mechanical or bioprosthetic valve replacement. At the time of prior surgeries, she preferred to avoid the teratogenicity and bleeding risks of warfarin during childbearing years, notably at the cost of eventual bioprosthetic valve degeneration. Now in her sixth decade, either mechanical or bioprosthetic valves would be reasonable.^1^ A mechanical mitral valve requires uninterrupted anticoagulation and a higher international normalized ratio goal of 2.5 to 3.5, which is nontrivial following a higher-risk cardiac surgery and for a patient who may be at increased fall risk. In contrast, with a bioprosthetic mitral valve, she would have lower bleeding risk on a direct oral anticoagulant.^4^^,^^5^ The size of aortic and mitral bioprosthetic valves that were chosen would be amenable to valve-in-valve transcatheter intervention should she require it in the future.

The patient's prolonged intensive care course was driven by RV failure postoperatively requiring inotropic and mechanical circulatory support. The etiology of impaired RV function was likely multifactorial including coronary ischemia, which was quickly recognized and treated with percutaneous coronary intervention. Repairing severe TR also carries the risk of acutely loading the right ventricle by removing its “pop-off valve,” particularly in the context of elevated pulmonary pressures. Prompt initiation of circulatory support and addressing drivers of RV failure minimized end-organ injury and ultimately helped the patient recover.

## Conclusions

Whereas this case demonstrates the feasibility of a complex multivalve operation performed by an experienced surgical team at a comprehensive valve center, it is also an example of the high morbidity that can follow. Multimodality imaging and hemodynamic data are often necessary to fully characterize valvular lesions before considering intervention. A patient-centered approach and multidisciplinary valve team involvement are vitally important for weighing treatment risks and developing a thorough care plan. Finally, having expertise to recognize and treat complications as well as involving specialty consultation are crucial to achieving successful outcomes in such complex cases.

**Visual Summary:**
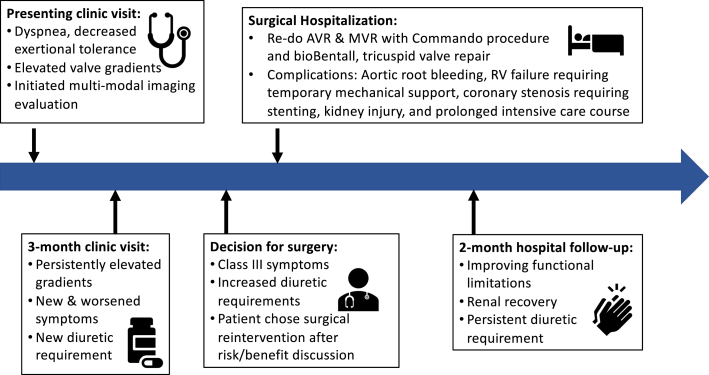
Timeline of Case Progression AVR = aortic valve replacement; MVR = mitral valve replacement; RV = right ventricular.

## Funding Support and Author Disclosures

The authors have reported that they have no relationships relevant to the contents of this paper to disclose.

## Appendix

Video 1 Cardiac Computed Tomography of Mitral Bioprosthetic Valve Cardiac computed tomography images in vertical long axis view showed mitral bioprosthetic valve leaflets are thickened and have abnormally reduced excursion in diastole, resulting in mitral stenosis.

Video 2 Mitral Bioprosthetic Valve on TEE Transesophageal echocardiography (TEE) from midesophageal view with color Doppler showed the mitral bioprosthetic valve leaflets with restricted motion and flow acceleration across the valve during ventricular diastole. There was mild to moderate mitral valve regurgitation.

Video 3 Leaflet Prolapse With Severe Aortic Bioprosthetic Valve Regurgitation The bioprosthetic aortic valve was challenging to visualize on echocardiography. Transesophageal echocardiography from mid-esophageal view in biplane with color Doppler showed the bioprosthetic valve in the aortic position with a flail cusp and severe regurgitation.

Video 4 Eccentric Aortic Valve Regurgitation on TEE Transesophageal echocardiography (TEE) midesophageal long axis view showed the bioprosthetic valve in mitral and aortic positions. The aortic bioprosthetic valve leaflets were not well seen in this view due to expected shadowing. However, color Doppler showed the eccentric aortic regurgitant jet directed posteriorly toward the anterior mitral leaflet, possibly further restricting mitral valve opening.
